# Gene Expression Changes of *Caenorhabditis elegans* Larvae during Molting and Sleep-Like Lethargus

**DOI:** 10.1371/journal.pone.0113269

**Published:** 2014-11-19

**Authors:** Michal Turek, Henrik Bringmann

**Affiliations:** Max Planck Research Group “Sleep and Wake”, Max Planck Institute for Biophysical Chemistry, Niedersachsen, Göttingen, Germany; Brown University/Harvard, United States of America

## Abstract

During their development, *Caenorhabditis elegans* larvae go through four developmental stages. At the end of each larval stage, nematodes molt. They synthesize a new cuticle and shed the old cuticle. During the molt, larvae display a sleep-like behavior that is called lethargus. We wanted to determine how gene expression changes during the *C. elegans* molting cycle. We performed transcriptional profiling of *C. elegans* by selecting larvae displaying either sleep-like behavior during the molt or wake behavior during the intermolt to identify genes that oscillate with the molting-cycle. We found that expression changed during the molt and we identified 520 genes that oscillated with the molting cycle. 138 of these genes were not previously reported to oscillate. The majority of genes that had oscillating expression levels appear to be involved in molting, indicating that the majority of transcriptional changes serve to resynthesize the cuticle. Identification of genes that control sleep-like behavior during lethargus is difficult but may be possible by looking at genes that are expressed in neurons. 22 of the oscillating genes were expressed in neurons. One of these genes, the dopamine transporter gene *dat-1*, was previously shown in mammals and in *C. elegans* to control sleep. Taken together, we provide a dataset of genes that oscillate with the molting and sleep-wake cycle, which will be useful to investigate molting and possibly also sleep-like behavior during lethargus.

## Introduction

Nematodes belong to the clade of ecdysozoans, which are protected against their environment by a cuticle [1]. In order to allow growth, the exoskeleton needs to get re-synthesized in a process called molting [2]. The nematode cuticle consists of a collagenous extracellular matrix that is synthesized by the hypodermis, an ectodermal tissue that is underlying the cuticle [2]. In a process called apolysis the old cuticle is separated from the hypodermis [2]. To synthesize a new cuticle, hypodermal cells and seam cells secrete, on their apical side, cuticle material that then hardens to form the new cuticle. The main component of the cuticle is collagen and the cuticle collagens form a large gene class with more than a hundred members [3]. In addition, cuticulins are cross-linking structural components of the cuticle[2], [4]–[6]. Gland cells secrete a surface coat that is rich in glycoproteins and lipids [2], [7]. After apolysis, in a process called ecdysis, worms shed the old cuticle [2], [8]. Ecdysis involves a typical shedding behavior [8] and depends on metalloproteases [9], [10].

Endocrine signals trigger regulatory cascades to express genes required for the molt [11]. The nuclear hormone receptor NHR-23 is required for the activation of many molting genes in the hypodermis [11], [12]. Genes that are downstream of NHR-23, such as the peptide MLT-8 and the Angiotensin Converting Enzyme ACN-1 were hypothesized to amplify the molting cue [11]. The production and secretion of cuticle-synthesizing proteins such as extracellular matrix components, peroxidases, proteases, and protease inhibitors leads to the formation of a new cuticle [11]. The repetition of these bursts of molting gene expression at the end of each larval stage causes an oscillating pattern of molting gene expression[13]–[17].

Circadian genes such as the *period* gene control circadian rhythms in other systems. *C. elegans* has a *period* homolog called *lin-42.* Similar to the *period* gene, *lin-42* mRNA levels oscillate with the molting cycle [15]. Knockout of *lin-42* causes defects in the timing of the molting cycle and the timing of the sleep-like behavior. Thus, circadian rhythm genes control the molting cycle in *C. elegans* [13].

During apolysis larvae do not feed and are behaviorally quiescent, a phenomenon called lethargus [18]. The quiescence behavior during lethargus fulfills behavioral criteria that define sleep [19], [20]. During lethargus, voluntary movement is largely absent[18], [20]–[22]. Behavioral quiescence can be reversed using sensory stimulation [20], [23], [24]. Quiescent larvae have an increased arousal threshold [20], [25]–[28]. Worms assume a specific body posture that is characterized by body wall muscle relaxation [22], [29]. Deprivation of quiescence demonstrated homeostatic regulation of this behavior [20], [24]. The quiescence behavior is controlled by the nervous system[22], [23], [25]–[28]. Because quiescence behavior during lethargus fulfills the behavioral criteria that define sleep in other species we call it sleep-like behavior.

Transcriptional changes underlie many biological processes. Previous work has found oscillating gene expression during molting and sleep in diverse species [14], [16], [17], [30]–[34]. The aim of this study was to investigate the changes in gene expression during the molting cycle in *C. elegans* larvae. We used behavioral criteria to determine whether animals were molting or not. We manually selected larvae during either sleep-like or wake behavior and analyzed the transcriptome using microarrays. Our analysis revealed 342 genes that were up regulated and 178 genes that were down regulated during molting. The majority of genes that were up regulated during lethargus seem to be related to the molting cycle. Only 22 of the oscillating genes are expressed in neurons but the majority of these genes have human homologs. One of these homologous genes, which encodes for a dopamine transporter, have been implicated in the control of sleep in mammals and in *C. elegans* [35], [36]. Our dataset should be useful for researchers that study the developmental and behavioral processes that occur during molting.

## Materials and Methods

### *C. elegans* maintenance

*C. elegans* was maintained on NGM plates at 21.5°C as described and all experiments were performed with wild type N2(Bristol) [37].

### Collection of *C. elegans* larvae during sleep-like and wake behavior

Gravid N2 hermaphrodites were bleached with hypochlorite solution to collect eggs, which were incubated in M9 buffer in a turn-over-turn rotator for 20 hours at room temperature. Arrested L1 nematodes were then placed on NGM plates seeded with *E. coli* strain OP50 and stored at 21.5°C. Between 36 and 38 hours later, when the majority of worms on the plates showed sleep-like behavior, 200–300 L3 lethargus animals were picked individually with a platinum wire pick and were immediately placed into a tube containing one ml of Trizol (Invitrogene). Two hours after the first collection, after worms had entered the L4 stage and were awake, the same number of worms was collected. The procedure was repeated for worms during L4 lethargus, and for young adults approximately two hours past the molt. To distinguish between worms in sleep- and in wake-like behavior, movement of the pharynx and the developmental stage of the vulva were examined for each individual. For each condition three biological samples were collected.

### Transcriptional profiling using microarrays

The Transcriptome Analysis Laboratory Göttingen (TAL) performed the Microarray analysis. The TAL used the “Low RNA Input linear Amplification Kit Plus, One Color” protocol (Agilent Technologies, Inc. 2007; Cat. N°: 5188–5339) and the Agilent RNA Spike-In Kit for One color (Agilent Technologies, Inc. 2007; Cat. N°: 5188–5282) following the manufacturer’s standard protocol. Global gene expression analysis was applied using the *C. elegans* (V2) Gene Expression Microarray, 4×44K from Agilent Technologies. The *C. elegans* (V2) Gene Expression Microarray contains 43,803 probes providing genome-wide coverage (more than 20,000 ORF). Some genes have multiple probes (AgilentIDs) designed for them, but not all different isoforms are covered.

200 ng of total RNA were used as a starting material to prepare cDNA. cDNA synthesis and in vitro transcription (IVT) were performed according to the manufacturer’s recommendation. Quantity and efficiency of the labeled amplified cRNA were determined using the NanoDrop ND-1000 UV-VIS Spectrophotometer version 3.2.1. The hybridizations were performed for 17 hours at 10 rpm and 65°C in the Hybridization Oven (Agilent). Washing and staining of the arrays were done according to the manufacturer’s recommendation. Cy3 intensities were detected by one-color scanning using an Agilent DNA microarray scanner (G2505B). Intensity data were extracted using Agilent’s Feature Extraction (FE) software (version 9.5.3.1) including a quality control based on internal controls using Agilent’s protocol GE1_107_Sep09. All chips passed the quality control and were analyzed using the Limma package of Bioconductor.

### Microarrays data analysis and statistics

The microarray data analysis was done as it was previously described [38]. Briefly, it consists of five steps: between-array normalization, cross correlation (hierarchical approach with the average linkage-method) and PCA-analysis, data fitting to a linear model, differential gene expression detection and GO term enrichment analysis.

To determine which genes are up or down regulated in L3 sleep-like state we subtracted gene expression levels between L3 sleep-like and L4 wake. Genes were counted as significantly up or down regulated for L3 sleep-like if the calculated false discovery rate (FDR) was equal or smaller then 5.0% and the fold change was >2. p-values were obtained from the moderated t-statistic and corrected with the Benjamini-Hochberg method. The same analysis was done for L4 sleep-like. To identify genes that are up or down regulated in both sleep-like phases, Venn analysis was performed. Oscillating genes were defined to have consistent expression changes between both lethargus and wake samples plus an expression change between L4 wake and L4 sleep-like of more than 50%. Phases of the oscillation for those genes were taken from a previous study [14] GO term enrichment analysis was done with DAVID Bioinformatics Resources 6.7 [39]. Only genes that were oscillating were included in this step.

For all of the microarrays probes that showed a significant change in the signal between the conditions, GenebankAccessions and GeneSymbols are provided in Table S1. Genes that did not have a GeneAccession info, were removed from the table.

### Quantitative reverse transcription PCR (RT-qPCR)

100 ng of total RNA were used as a starting material to prepare cDNA. Reverse transcription was performed on a FlexCycler^2^ thermocycler using the High-Capacity cDNA Reverse Transcription Kit (Applied Biosystems) according to the manufacturer’s recommendation. qPCR was done on a StepOne Plus thermocycler using the Fast SYBR Green Master Mix (Applied Biosystems) following the manufacturer’s protocol. Reactions for all genes were run in technical triplicates. Ct values for *dat-1* and *inx-19* were corrected for *act-1* expression. Primers used were as follows: *act-1* forward gttgcccagaggctatgttc, *act-1* reverse caagagcggtgatttccttc (taken from a previous study [14]), *inx-19* forward tggagactctgcagctttca, *inx-19* reverse ttccgttcggagattgtagg, *dat-1* forward ctcaggcgcctcatttattc, *dat-1* reverse tgtttgattcggcctttttc (designed with the Primer3 software [40]).

## Results

### Identification of genes with altered expression during molting

**Figure 1:**
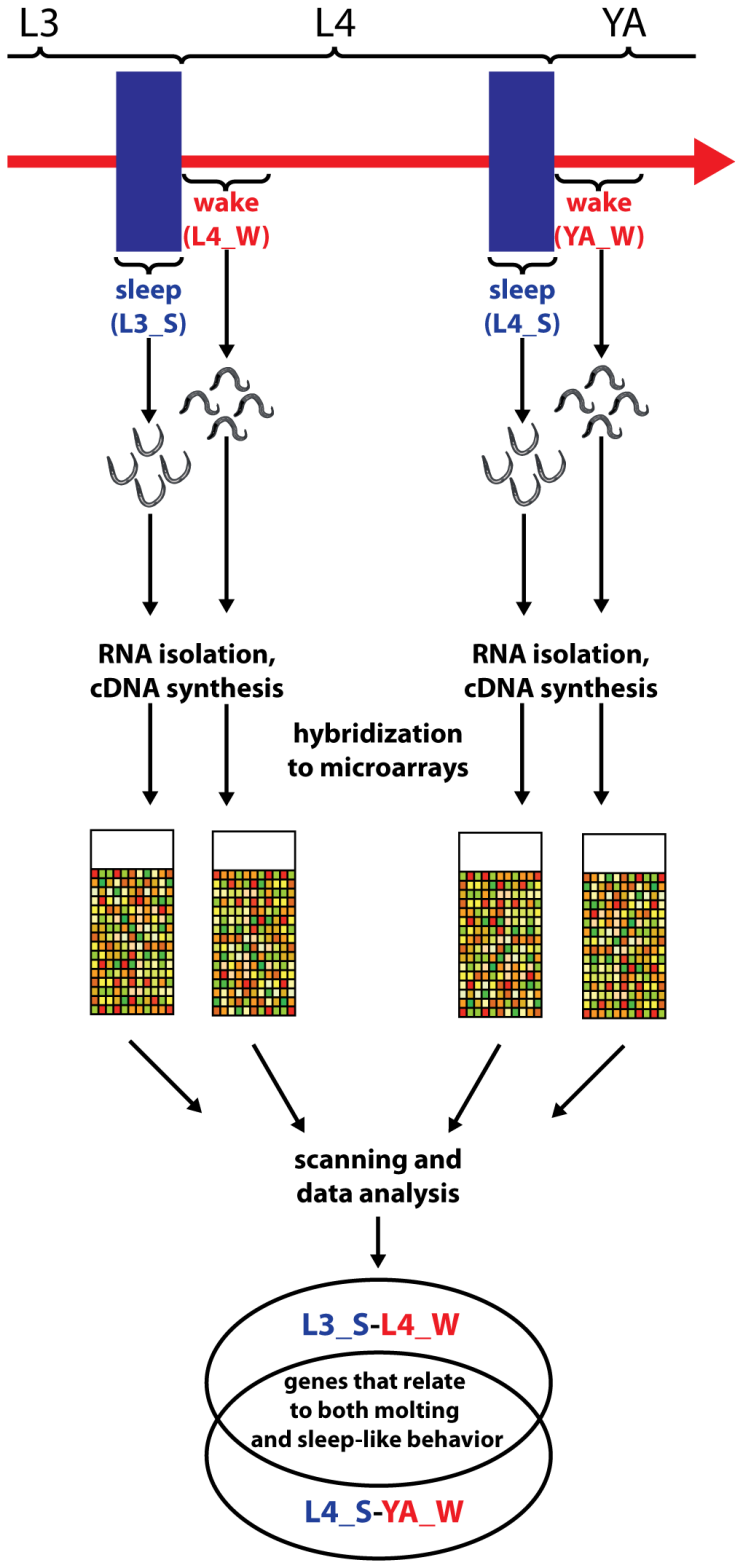
Strategy of the transcriptional profiling to identify genes that are enriched during molting and lethargus. Strategy of the transcriptional profiling: Worms were individually staged using behavioral criteria, namely sleep-like and wake behavior, and subjected to microarray analysis. Because sleep-like behavior in larvae occurs during the molt, we obtained a dataset that reflects the expression changed during the molt and sleep-like behavior. Worm images are from [23].

**Figure 2:**
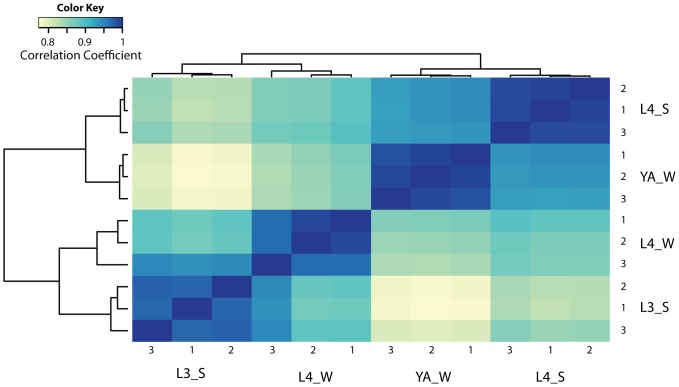
Cross-correlation analysis. Cross-correlation plot obtained by microarray analysis of worms during L3 sleep-like behavior, L4 wake, L4 sleep-like behavior, and young adults during wake. Heat map analysis shows that the biggest changes in gene expression are due to developmental progression and not molting.

**Figure 3:**
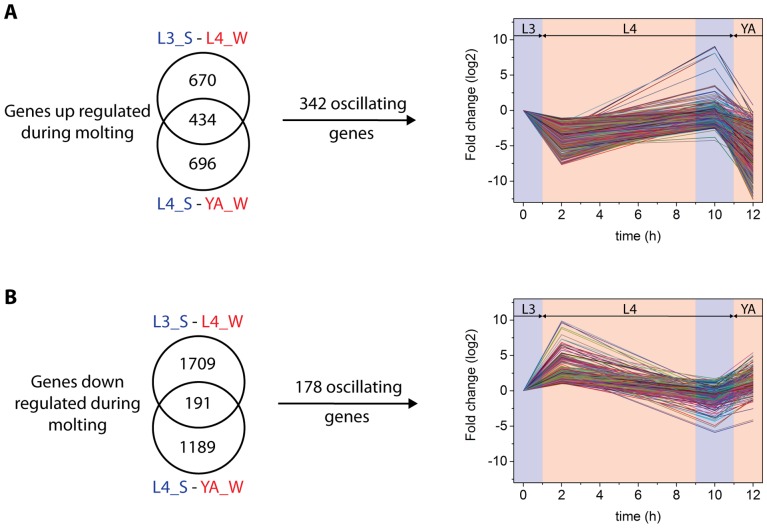
Genes that oscillate with the molting cycle. A) Genes that are up regulated during molting and that show an oscillation with the molting cycle with an amplitude of at least 50%. B) Genes that are down regulated during molting and that show an oscillation with the molting cycle with an amplitude of at least 50%. Time “zero” corresponds to 36 to 38 hours after the release of L1 from starvation. The L4 stage lasts about 10 h. Lethargus animals were collected around timepoint 10 h for a period of 2 h based on the behavior of the worms. Only animals that were not pumping and not moving were collected.

We wanted to identify genes that have altered expression during molting. We visually identified the behavioral state of individual worms from a synchronized population of worms and cherry picked them for transcriptional analysis. We defined molting by the concomitant sleep-like behavior that is characterized by the absence of feeding for more than 20 seconds. To reduce unspecific changes in transcription due to developmental progression we identified worms outside of the molt only in a short time window of up to about two hours after the sleep-like behavior, when worms were pumping again (Figure 1). We wanted to identify core genes that are altered in all lethargus phases and thus wanted to exclude genes that are specific for only one larval stage. To achieve this goal, we compared two lethargus phases at two different larval stages and selected genes that had altered expression levels in both lethargus phases. We selected four different conditions containing two different stages of post-molting and two different stages of molting: We used molting L3 (L3_S), post-molting L4 (L4_W), molting L4 (L4_S), and post-molting young adults (YA_W) (Figure 1). Worms were manually transferred into Trizol solution and were thus killed immediately. mRNA was extracted and transcriptional profiles were obtained using Agilent arrays. Cross-correlation analysis revealed that L3 molting worms and L4 post-molting worms were more similar than L4 post-molting worms and L4 molting worms. Similarly, L4 molting worms and post-molting young adult wake worms were more similar than L4 post-molting worms and L4 molting worms. The biggest difference was between L3 molting worms and post-molting young adult wake worms. There was a moderate similarity between L3 and L4 molting worms and between L4 and young adult post-molting worms (Figure 2). This result suggests that the largest determinant of similarity in gene expression is developmental progression through different larval stages and not molting itself. This result is consistent with the large change in gene expression during development that was recently reported [14], [16]. Comparison of L3 molting worms with L4 post-molting worms revealed 1104 genes that were up regulated during molting. Comparison of L4 molting worms with post-molting young adults revealed 1130 genes that were up regulated during molting. Of these genes, a set of 434 genes was up regulated in both the L3 and L4 molt (Figure 3, Table S1). The same comparison revealed that 1900 genes were down regulated during L3 molting compared with L4 wake and that 1380 genes were down regulated during L4 molting compared with young adult post-molting. Of these genes, a set of 191 genes was down regulated in both the L3 and L4 molt (Figure 3, Table S1). We reasoned that gene expression changes that would be biologically relevant would oscillate with substantial amplitude in phase with the molting cycle. We applied an arbitrary threshold and selected only those genes that oscillated with an amplitude of at least 50%. We obtained a core set of genes that oscillated with the molting cycle of 342 genes that were up regulated during molting and 178 genes that were down-regulated during molting (Figure 3, Table S1). For some of the genes, changes in expression levels were quite huge: 139 genes that were up regulated during molting and 17 genes that were down regulated during molting showed a more than ten fold change in expression. Thus, our transcriptional profiling revealed 342 oscillating core genes that are up regulated during molting and 178 oscillating core genes that are down regulated during molting.

### Validation of data by comparison to known oscillating genes and by qPCR

**Figure 4:**
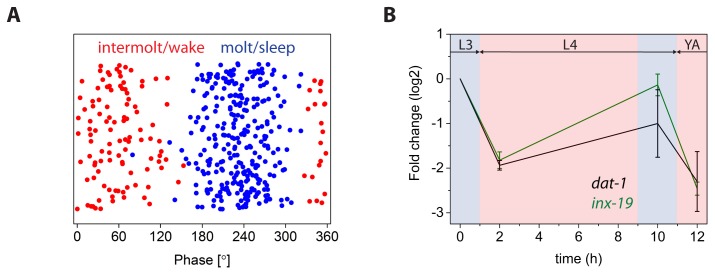
Validation of dataset. **A)** Oscillation phase of genes that have altered gene expression during molting. Comparison of oscillating genes with previously published data [14] validates the data set. Each point represents one gene that was shown to oscillate both in previously published data and in our data set. **B)** qPCR analysis confirms oscillation of expression of *inx-19* and *dat-1*. Time “zero” on x axis corresponds to 36 to 38 hours after the release of L1 from starvation.

Recent work used RNA sequencing to show that there is extensive oscillatory gene expression during *C. elegans* larval development [14], [16]. We wanted to validate our data set and see how many of the genes that we have identified have been reported to be oscillating. We found that from the 178 core genes that were down regulated during molting 128 were shown to be oscillating according to the literature [14]. From the 342 core genes that were up regulated during molting 254 were shown to be oscillating [14] (Figure 4). We then looked at the phase of the oscillation of the expression of the genes that were both previously shown to be oscillating and that we also identified to be enriched during molting. The phase is defined by dividing the length of the full period, roughly eight hours, by 360°. This results in 45° corresponding to one hour. The phase for each gene is determined by fitting a cosine function with a fixed period of eight hours [14]. Almost all genes that we found to be enriched during molting had a phase of about 170°–290° (p = 2.9*10^−94^), and all genes that were enriched during post-molting were outside of this phase range (p = 6.5*10^−23^). This indicates that we were able to identify oscillating genes that have the peak oscillation during the molt. Thus, our dataset obtained for molting and post-molting worms using microarrays is consistent with data obtained for developmentally staged worms using RNA sequencing [14]. In addition to the previously identified oscillating genes we found 88 new genes with oscillating expression that are up regulated during molting, and 50 new genes with oscillating expression that are down regulated during molting.

To further validate the oscillation of expression, we performed qPCR for two genes that were not shown previously to oscillate, *inx-19* and *dat-1*. Both genes showed oscillating gene expression according to qPCR analysis consistent with the microarray results (Figure 4B). Thus, we provide a validated data set of novel oscillating genes.

### Genes that are up regulated during molting

342 genes were up regulated during molting. To identify biological processes relevant to molting we performed a gene ontology term (GO term) enrichment analysis (Table 1). This analysis showed that the vast majority of genes that we found appear to relate to cuticle synthesis and not to the sleep-like behavior, consistent with previous results [14].

**Table 1:** GO term enrichment and depletion analysis.

GO term^1^	Number of genes	P-value	Fold change
**Up regulated**			
structural constituent of cuticle (MF)	19	1.80E-12	9.1
structural molecule activity (MF)	22	9.20E-07	3.5
extracellular region (CC)	18	1.20E-06	4.2
serine-type endopeptidase inhibitor activity (MF)	9	7.30E-06	8.8
response to stress (BP)	11	3.20E-04	4.1
membrane (CC)	113	4.60E-04	1.2
metallopeptidase activity (MF)	10	5.10E-04	4.3
chitin metabolic process (BP)	4	2.40E-03	14.5
defense response (BP)	4	2.90E-02	5.9
amine transport (BP)	3	3.00E-02	10.9
peroxidase activity (MF)	3	4.40E-02	8.9
**Down regulated**			
catalytic activity (MF)	57	4.30E-07	1.7
metabolic process (BP)	51	1.30E-04	1.5
transferase activity (MF)	25	1.60E-04	2.2
lipid metabolic process (BP)	10	1.60E-03	3.6
L-serine metabolic process (BP)	3	2.10E-03	41.6
cation binding (MF)	30	2.30E-03	1.7
carboxylic acid biosynthetic process (BP)	4	1.50E-02	7.7
monooxygenase activity (MF)	5	1.50E-02	5.2
integral to membrane (CC)	68	2.10E-02	1.2
icosanoid metabolic process (BP)	2	2.60E-02	74
cysteine synthase activity (MF)	2	3.50E-02	55.6
ecdysis, collagen and cuticulin-based cuticle (BP)	2	4.40E-02	44.4
cellular ketone metabolic process (BP)	6	5.00E-02	3

1 MF – molecular function, CC – cellular component, BP – biological process. The P-value was calculated with a Fisher’s Exact test (modified as EASE score). The P-value gives an indication how likely it is that a certain GO term is represented by a given number of genes (coming from the analysis) by chance. Compared to the total number of genes representing certain GO term in the whole *C. elegans* genome.

### Genes with neuronal expression that oscillate with the molting cycle

The majority of genes that oscillate with the sleep-wake-like cycle appear to relate to molting. In order to obtain a starting point for investigating the behavioral changes during lethargus we selected those genes that both oscillated and are expressed in the nervous system [41]. We found only 18 neuronal genes that were up regulated during sleep-like behavior and four genes that were down regulated during sleep-like behavior (Table 2). This de-enrichment of neuronal genes is consistent with a previous analysis [14]. The majority of the neuronal oscillating genes have human homologs. Among the genes that were up regulated there were two G-protein coupled receptors (B0334.6, Y70D2A.1), insulin-like molecules and a neuropeptide (*nlp-24*, *ins-18*, *ins-21*), ligand-gated ion channels (*lgc-18*, *lgc-21*, *lgc-31*), calcium-binding protein genes that contain EF hands (F23F1.2, H10E21.4, K03A1.4), serpentine/seven transmembrane helix receptors (*srz-78*, *str-131*, *str-146*) and the dopamine plasma membrane transporter gene *dat-1*. Among the genes that were down regulated during sleep-like behavior were a carboxypeptidase gene (*cpd-1*), dipeptidyl peptidase IV gene *dpf-6* and a calcium-binding protein gene that contain EF hand (Y12A6A.1). Only one of these genes, *dat-1*, has been implicated in the control of sleep-like behavior in *C. elegans* [36]. Dopamine transporters have also been implicated in the control of sleep in mammals and are the target of the wakefulness-inducing drug modafinil [35]. Thus, our dataset of neuronal oscillating genes presents a starting point for studying sleep-like behavior in *C. elegans*.

**Table 2:** Genes that oscillate with the molting cycle and that are expressed in neurons.

Gene name	Putative gene role	Close human homolog	Putative homolog role
**Up regulated**			
B0334.6	probable G-protein coupled receptor	GPR139 (89.1%)	probable G-protein coupled receptor
daf-10	intraflagellar transport	IFT122 (74.7%)	intraflagellar transport protein
dat-1	dopamine transporter	SLC6A2 (89.3%)	noradrenaline transporter
F23F1.2	EF-hand Ca^2+^ binding	–	–
H10E21.4	EF-hand Ca^2+^ binding	CALM1 (50.0%)	calmodulin
ins-12	insulin-like molecule	–	–
ins-20	insulin-like molecule	–	–
inx-19	innexin (gap junction protein)	–	–
K03A1.4	EF-hand Ca^2+^ binding	cDNA FLJ75174 (76.1%)	highly similar to calmodulin
lgc-18	ligand-gated ion channel	CRNA1 (77.9%)	associated with cholinergic receptor
lgc-21	ligand-gated ion channel	–	–
lgc-31	ligand-gated ion channel	ENSP00000347754 (54.1%)	5-hydroxytryptamine receptor
lim-8	links membrane attachmentproteins to myosin thick filaments	LMO7 (74.8%)	-
nlp-24	neuropeptide	ENSP00000380734 (66.2%)	-
srz-78	serpentine receptor	–	–
str-131	seven TM receptor	–	–
str-146	seven TM receptor	–	–
Y70D2A.1	probable G-protein coupled receptor	GPR139 (56.0%)	probable G-protein coupled receptor
**Down regulated**			
cal-3	EF-hand Ca^2+^ binding	CALM1 (61.1%)	calmodulin
cpd-1	carboxypeptidase D	CPD (60.8%)	carboxypeptidase D
dpf-6	dipeptidyl peptidase four (IV)	APEH (31.1%)	acylamino-acid-releasing enzyme
Y12A6A.1	–	–	–

## Discussion

Molting and sleep have been shown in several species to be accompanied by changes in gene expression[14], [16], [17], [30]–[34]. Here we find that molting, which is characterized by re-synthesis of a new cuticle and sleep-like behavior, also is accompanied by changes in gene expression. Because synthesis of a new cuticle is a biochemically demanding process it is not surprising that the majority of genes for which we found altered gene expression levels appear to relate to the molting process [2]. This result is consistent with previous reports [14].

Because of the vast number of genes that are required for synthesizing a new cuticle, it is difficult from this analysis to predict which, if any, of the genes are required for the behavioral changes during lethargus such as the sleep-like behavior. We preselected potential regulators of sleep-like behavior by selecting only genes that are expressed in neurons. Most of these oscillating neuronal genes have homologs in mammals. This dataset contains at least one gene, *dat-1*, which has been implicated in the control of sleep [35], [36]. The neuronal oscillating genes provide a potential starting point for investigating the genetics of sleep-like behavior.

Our analysis revealed novel oscillating genes but it also did not re-identify all genes that were previously shown to oscillate. Prominent examples of oscillating genes that we did not find are *lin-42* [15] and *mlt-10* [11]. A potential explanation for why our dataset lacked these genes can be found by looking of the phases of these genes, which are 100° and 136°, respectively. Our analysis aimed to identify genes with altered expression during molting, not all genes that have oscillating expression across development. We thus sampled worms during molting (corresponding to approx. 170°–290°) and two hours post molting (corresponding to approx. 330°–60°). Thus, our analysis covers molting well, but does not cover all phases of the intermolt. As can be seen in Figure 4, we did not find many hits in the phase range of 120° to 170°. *lin-42* and *mlt-10* peak in this area, which may explain why we have not identified them. Because the worms were selected based on a behavioral phenotype the population of selected animals could be enriched in core lethargus animals. Sleep-like immobility is most prominent during the middle of the lethargus period. Thus, our data set may not contain many animals from the early or late phase of lethargus and genes that have maximum mRNA expression during this time may have been missed. Thus, while we think our dataset effectively identified genes that are enriched during molting it did not identify all genes that oscillate during development.

Here we provide a dataset that includes many genes that were not previously shown to oscillate with the molting cycle. The data should be useful for researchers that are working on changes that occur during molting.

## Supporting Information

Table S1 **Genes with altered expression during sleep-like and wake behavior.**
(XLSX)
